# Clinical predictor of survival following docetaxel-based chemotherapy

**DOI:** 10.3892/ol.2014.2349

**Published:** 2014-07-14

**Authors:** HSIANG-YING LEE, WEN-JENG WU, CHUN-HSIUNG HUANG, YII-HER CHOU, CHUN-NUNG HUANG, YUNG-CHIN LEE, KAI-FU YANG, MEI-HUI LEE, SHU-PIN HUANG

**Affiliations:** 1 Department of Urology, Kaohsiung Medical University Hospital, Kaohsiung 807, Taiwan, R.O.C; 2 Department of Urology, Kaohsiung Municipal Hsiao-Kang Hospital, Kaohsiung 812, Taiwan, R.O.C; 3 Department of Urology, College of Medicine, Kaohsiung Medical University Kaohsiung, Kaohsiung 807, Taiwan, R.O.C

**Keywords:** castration-resistant prostate cancer, docetaxel

## Abstract

Prostate cancer (PCa) is the most common type of cancer in males in the USA and the incidence is increasing. For castration-resistant PCa (CRPC), previous studies have identified docetaxel-based chemotherapy as the first-line therapy. In the present study, the efficacy of docetaxel-based chemotherapy was investigated in a population of patients with CRPC. This study included 26 individuals (mean age, 73 years) with CRPC who were patients between July 2007 and October 2012 at the Kaohsiung Medical University Hospital (Kaohsiung, Taiwan). The regimen consisted of intravenous docetaxel (70 mg/m^2^) once every four weeks plus oral prednisolone (5 mg) twice daily for five days. Prostate-specific antigen (PSA) response (defined as a PSA decrease of >50% over four weeks), time to PSA progression, PCa-specific survival and overall survival (OS) were evaluated. For these 26 patients, the mean PSA level prior to chemotherapy treatment was 335.58 ng/ml. During follow-up, the average number of cycles of chemotherapy was approximately seven and 15 patients (58%) achieved a PSA response. PSA response was found to significantly correlate with OS and PCa-specific survival (P=0.014 and P=0.028, respectively). The mean value of the PSA nadir level was 89.97 ng/ml and time to PSA nadir was five months. The most common adverse event was leucopenia, which affected 88% of the patients. The results indicated that the length of time to PSA nadir and the occurrence of leucopenia may impact the PSA response. The docetaxel-based chemotherapy was a feasible and effective treatment regimen in patients with CRPC. However, the occurrence of adverse events, particularly the high incidence of leucopenia, may be cause for concern.

## Introduction

Prostate cancer (PCa) is the most common type of malignancy and the second leading cause of cancer mortality in males in Western countries (1). In the USA in 2011, there were 240,890 new cases of PCa diagnosed and 33,720 PCa-specific mortalities. In Taiwan, PCa is the seventh most common cause of cancer-associated mortality and the incidence is increasing, particularly in elderly populations. Due to the widespread implementation of prostate-specific antigen (PSA) screening, the earlier detection of PCa has increased. Although the incidence of PCa has rapidly increased in Taiwan (30 cases per 100,000 individuals in 2010), this is significantly lower than that of the USA and Europe (2). When the PCa is not metastatic, it can be treated with radiotherapy or surgery; however, the treatment of metastatic PCa is palliative. PCa initially grows in an androgen-dependent manner, and therefore, androgen deprivation therapy (ADT) may improve symptoms and reduce PSA levels in ~80% of metastatic PCa patients (3). After an average of two years undergoing hormone therapy, the majority of metastatic PCa cases are likely to develop into castration-resistant PCa (CRPC) (4). Chemotherapy for CRPC was implemented for a number of years, although it had limited efficacy, until two landmark randomized control trials were reported. Once complete, the two phase III trials demonstrated that a docetaxel-based combined chemotherapy regimen may improve survival rates in CRPC patients (3,4). In the TAX 327 study, the median overall survival (OS) time was improved in the docetaxel plus prednisone treatment group compared with the mitoxantrone plus prednisone treatment group (3). In the Southwest Oncology Group (SWOG) study, the docetaxel plus estramustine regimen showed a survival benefit versus the mitoxantrone plus prednisone regimen (5). Therefore, in 2004, docetaxel was approved by the European Medicine Agency and the US Food and Drug Administration as the first-line treatment for patients with CRPC. The aim of the present study was to retrospectively evaluate the efficacy and toxicity of treatment with docetaxel plus prednisolone every four weeks in patients in Taiwan with CRPC, as there are few studies of this type concerning Asian populations.

## Patients and methods

### Patients

A total of 26 patients with CRPC were reviewed retrospectively between July 2007 and October 2012 at the Kaohsiung Medical University Hospital (Kaohsiung, Taiwan). All patients provided written informed consent. This study was approved by the institutional review board of Kaohsiung Medical University Chung-Ho Memorial Hospital (Kaohsiung, Taiwan). The eligibility criteria included histopathologically diagnosed progressive prostate adenocarcinoma with disease progression despite ADT and no previous chemotherapy.

### Treatment

All patients received docetaxel plus prednisone therapy with or without estramustine. The regimen consisted of docetaxel (70 mg/m^2^), which was administered over 60 min through intravenous infusion once every four weeks, plus oral prednisolone (5 mg) twice daily for five days (days one to five). All cases were defined as CRPC according to the European Association of Urology guideline 2011 (6). Serum PSA levels were measured every four weeks during treatment. When each course was initiated, a complete blood count was performed, and renal and liver function were assessed. The time to PSA progression, PCa-specific survival, OS and the PSA response of all patients were evaluated. The OS rate was measured as the time between the initial docetaxel administration and mortality. The definition of PSA response was a reduction of ≥50% from the baseline levels for at least four weeks. PSA progression was defined as a >50% increase from the PSA nadir and an increase in the absolute PSA level by ≥5 ng/ml with confirmation following at least one week (7). The PSA nadir was defined as the lowest PSA level achieved during treatment. The time to PSA progression was assessed between the day of treatment initiation and PSA progression. Treatment with docetaxel was continued until disease progression, unacceptable adverse effects, or the refusal of the patient to receive further therapy.

The National Cancer Institute Common Toxicity Criteria version 4.0 was used to evaluate the toxicity during every cycle (8). Granulocyte colony-stimulating factor (G-CSF) was administered when grade 3 or 4 leucopenia and febrile leucopenia occurred according to physician’s experience. The definition of leucopenia was a white blood cell count of <4,400/μl.

### Statistical analysis

All analyses were performed using SPSS software, version 19.0 (IBM, Armonk, NY, USA). The probability of PCa-specific survival and OS between PSA response and no response was calculated using Kaplan-Meier analysis. Following the univariate analysis, a multivariate analysis was performed to elucidate the effect of these combined variables. P<0.05 was considered to indicate a statistically significant difference.

## Results

### Patient characteristics

The characteristics of 26 patients with CRPC are summarized in Table I. In the present study, prior to the introduction of docetaxel-based chemotherapy, the mean serum PSA level was 335.58±501.87 ng/ml (normal range, <20 ng/ml) and the mean serum hemoglobin (Hb) level was 10.70±1.69 g/dl (normal range, 14–17 g/dl). During the period of chemotherapy, a mean number of cycles (6.92±3.03) were administrated and four patients received >10 cycles.

### PSA response

A PSA decrease of >50% from the baseline was observed in 15 (58%) of the 26 patients. During the treatment, the mean PSA nadir level was 89.97±117.76 ng/ml and the time to PSA nadir and progression was 4.65±3.60 and 6.50±5.85 months, respectively. No significant differences were identified between certain variables, including age, Hb level, PSA level, PSA nadir, PSA decline and the number of cycles of estramustine, in the PSA response and non-PSA response groups in Table II. However, the results indicated that the occurrence of leucopenia may be associated with a higher PSA response rate (P=0.032). In addition, the length of the time to PSA nadir was shown to significantly affect PSA response. For the variation in PSA kinetics, the PSA decline was calculated using an algorithm according to the following formula: PSA decline = Log [(PSA level prior to chemotherapy - PSA nadir)/survival time (months)]. The results of univariate and multivariate Cox regression analyses for the OS rate of 26 patients are summarized in Table III. The univariate and multivariate analyses revealed that the number of chemotherapy cycles and time to PSA nadir were independent prognostic factors of the OS rate (P<0.05).

### Kaplan-Meier analysis

**Figure 1:**
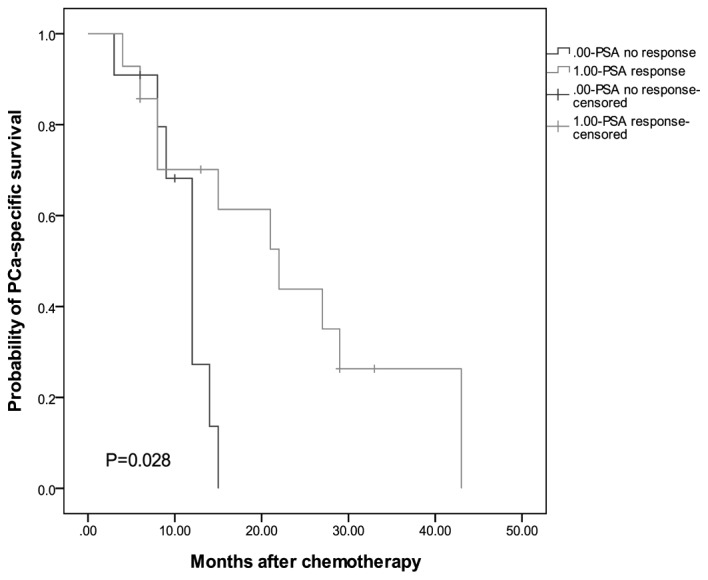
Kaplan-Meier estimates of prostate cancer-specific survival according to PSA response status. PSA, prostate-specific antigen. P<0.05.

**Figure 2:**
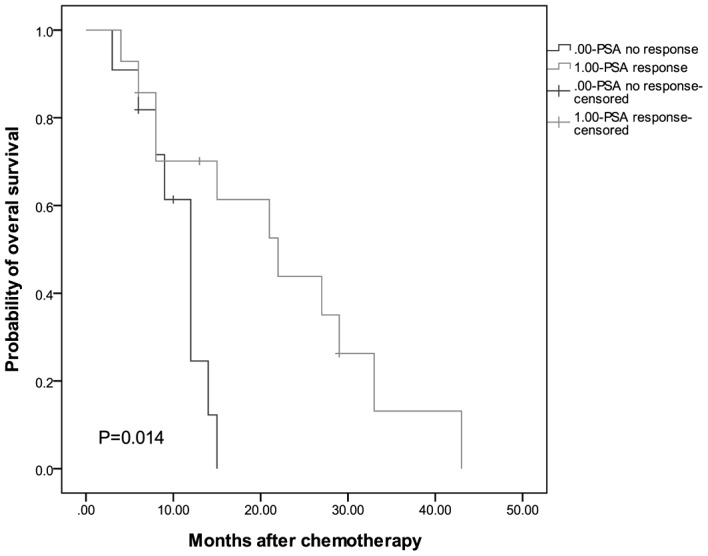
Kaplan-Meier estimates of overall survival according to PSA response status. PSA, prostate-specific antigen. P<0.05.

As shown in Figs. 1 and 2, in order to identify the possible prognostic factors of docetaxel-based chemotherapy, the correlation between certain factors and survival rates was analyzed. The results indicated that PSA response is a significant predictor of OS (P=0.014) and PCa-specific survival (P=0.028).

## Discussion

According to two large, randomized trials (3,5), docetaxel-based chemotherapy is currently the standard chemotherapy for CRPC due to its survival benefit. The present study retrospectively assessed the oncologic outcomes of 26 patients with CRPC to identify prognostic factors. At the Kaohsiung Medical University Hospital, a docetaxel-based chemotherapy regimen, consisting of 70 mg/m^2^ docetaxel once every four weeks and 5 mg prednisolone twice daily on days one to five for each cycle, was administered, which is a different protocol compared with that of previous studies (3,5,7). Clinically, it is common to add steroids to docetaxel-based chemotherapy in CRPC. The mechanism of the combination of docetaxel and prednisolone may increase the cytotoxic efficacy and antiangiogenic activity of docetaxel by steroids (9).

In the present study, the PSA response was 58%, which was similar or superior to the results observed in previous studies. For example, Hideaki *et al* (10) reported a PSA response rate of 55.6%. In addition, Naito *et al* (11) and Lee *et al* (12) reported PSA response rates of 44.4% and 51%, respectively. The median OS time of the current patients following chemotherapy was 14.84 months, which was shorter than that observed in the two landmark studies (19.2 months in the TAX327 study and 17.5 months in the SWOG 99-16 study). However, the mean PSA levels prior to chemotherapy in these two studies (114 ng/ml in the TAX327 study and 84 ng/ml in the SWOG 99-16) were lower than those identified in the current study. The results indicated that the survival time is associated with pretreatment PSA levels. Although, different patient characteristics and the small sample size may also be influencing factors. Further comparisons are required between the various therapy periods to assess whether a duration of four weeks is more effective than that of three weeks.

Furthermore, the results demonstrated that PSA response is an important predictor factor of OS and PCa-specific survival rates. However, it is important to be aware of the PSA flare-up phenomenon, which leads to a transient PSA elevation following chemotherapy (13). In the current study, two patients exhibited an increase in their PSA levels on initiation of treatment, which was followed by a decline in PSA levels after five weeks (Table IV). This may occur between one and eight weeks following a drop in the level of serum PSA (14). There is no firm hypothesis for the mechanism of post-chemotherapy PSA surge syndrome. However, one theory proposes that PSA is released on the destruction of cancer cells or the increase may be due to local inflammation (15,16). Therefore, chemotherapy may be continued for at least three cycles to avoid inadequate therapy, provided no severe side effects develop.

Although 10 cycles of docetaxel are generally used as the standard treatment and a recent study has concluded that >10 cycles of docetaxel-based chemotherapy for CRPC does not improve overall survival, in the present study, treatment was continued for >10 cycles (17). The number of cycles administered was based on the patient’s medical condition and disease progression. Of the 26 patients, four received >10 cycles of treatment and these four patients all exhibited a PSA response and had survival times of more than two years.

The most common adverse effect was leucopenia and this affected 88% of patients in the present study. Of these patients, four cases of febrile leucopenia were identified, and antibiotics were administered in these instances. The incidence of leucopenia was higher than that observed in Western populations (7). There has been an assumption that the polymorphisms of CYP3A isoenzymes cause the difference between ethnic backgrounds (7). In the current study, the white blood cell count was measured prior to and following chemotherapy in the patients that exhibited an associated infection. If patients had fever or grade 3 or 4 neutropenia, the administration of G-CSF was proposed. Among these cases, interstitial pneumonitis was observed in one patient, who presented with a fever, a cough with sputum, dyspnea and diffuse lung infiltrates. Although chemotherapy were discontinued, the patient succumbed as a result of respiratory failure. Previous studies have demonstrated that the incidence of interstitial pneumonitis is ~0.2% and it is considered that this may result from docetaxel administration (7,16). In the majority of cases, patients recover following the discontinuation of docetaxel-based chemotherapy; however, it can be lethal, as observed in the current study. In cases of severe interstitial pneumonitis, lung function is examined prior to commencing chemotherapy, particularly on elderly patients or those with a history of lung disease. In addition, luteinizing hormone-releasing hormone agonists and non-steroidal anti-androgens may also cause interstitial pneumonitis; the latency of which may persist for up to eight months (18). Prior to the onset of CRPC, all patients in the present study received hormone therapy, thus, clinicians must aim to prevent vulnerable patients from developing interstitial pneumonitis. The results of the current study indicated that the onset of leucopenia may be associated with PSA response. The assumption is that the more susceptible the PCa is to treatment with docetaxel, the more susceptible the patient is to adverse effects.

In total, 12 of the 26 patients received concomitant estramustine therapy, which appeared to provide no survival benefit, however, the small sample size may not be representative. It has been proposed that the addition of estramustine is likely to increase the risk of cardiovascular events, including thrombosis and myocardial infarction. Although no associated lethal adverse effects were observed during this study, this possibility cannot be ignored, and prophylactic anticoagulant agents may therefore be prescribed to patients who have a history of, or who are at risk of, cardiovascular toxicity (19). However, Qi *et al* (20) concluded that the survival rates with regard to PSA response rate were not improved with the addition of estramustine to docetaxel-based chemotherapy (4). Therefore, the toxicity profile must be considered prior to the addition of estramustine to the therapeutic regimen.

Multivariate analysis of the prognostic factors revealed a significant correlation between OS rates, and the number of chemotherapy cycles and time to PSA nadir. The results revealed that an increased number of treatment cycles and longer time to PSA nadir correlate with a longer OS. Therefore, time to PSA nadir may represent an adaptive ability. However, the mechanism of association requires confirmation; a significant heterogeneity in CRPC may lead to this result (21). Previous studies have evaluated prognostic factors as a method of predicting survival outcomes for CRPC patients prior to chemotherapy. The retrospective analysis presented by Qu *et al* (22) showed that the measurement of PSA doubling time, baseline Hb concentration, baseline alkaline phosphatase concentration, number of chemotherapy cycles and time to castration resistance, may provide predictive information. However, due to the retrospective nature and small sample size of the current study, future prospective studies with larger populations of patients are required.

In conclusion, docetaxel and prednisolone combination chemotherapy was an effective treatment regimen in Taiwanese CRPC patients, with high rates of PSA response and improved survival rates. The most common side effect was leucopenia, which can be controlled by administration of G-CSF. However, a careful assessment prior to therapy is required for the prevention of lethal adverse effects, including interstitial pneumonitis and cardiovascular events. This retrospective study demonstrated that PSA response, number of chemotherapy cycles and time to PSA nadir may provide prognostic factors for OS. However, further prospective studies investigating a larger population are required to evaluate more prognostic factors within Asian populations.

## Figures and Tables

**Table I:** Characteristics of the 26 CRPC patients.

Characteristic	Value
Age, years	
Mean ± SD	73.19±9.93
Range	54.00–88.00
Cycles, n	
Median ± SD	6.92±3.03
Range	2.00–14.00
Serum PSA, ng/ml	
Mean ± SD	335.58±501.87
Range	14.67–2363.56
Hemoglobin, g/dl	
Mean ± SD	10.70±1.69
Range	7.50–13.70
Concomitant estramustine, n (%)	12 (46)
Site of metastasis, n (%)	
Bone	25 (96)
Lymph node	8 (31)
Liver	1 (4)
PSA response, n (%)	15 (58)
No PSA decrease, n (%)	3 (12)
PSA nadir, ng/ml	
Mean ± SD	89.97±117.76
Range	0.84–448.59
Time to PSA nadir, months	
Mean ± SD	4.65±3.60
Range	0.50–13.00
Time to progression, months	
Mean ± SD	6.50±5.85
Range	0.00–23.00
Survival time following chemotherapy, months	
Mean ± SD	14.69±10.14
Range	3–43
Mortality, n (%)	20 (77)
Side-effect, n (%)	
Leucopenia	23 (88)
Febrile leucopenia	4 (15)

PSA, prostate-specific antigen; SD, standard deviation.

**Table II:** Associated factors between PSA response and PSA no response.

Factor	PSA response (n=15)	PSA no response (n=11)	P-value
Age, years	72.33±9.68	74.36±10.61	0.616
Hemoglobin, g/dl	10.54±1.57	10.93±1.89	0.574
PSA level, ng/ml	424.38±625.90	214.48±230.83	0.301
PSA nadir, ng/ml	63.93±87.27	138.8±155.47	0.151
Time to PSA nadir, months	5.77±3.77	2.56±2.13	0.039
PSA decline^a^	0.17±0.11	0.19±0.13	0.787
Cycles, n	7.80±3.51	5.73±1.74	0.061
Leukopenia, n (%)			0.032
Yes	15 (65.20)	8 (34.80)	
No	0 (0.00)	3 (100.00)	
Estramustine, n (%)			0.391
Yes	8 (66.70)	4 (33.30)	
No	7 (50.00)	7 (50.00)	

a PSA decline = Log [(Pre-chemotherapy PSA - PSA nadir)/Survival time] where the units are as follows: PSA, ng/ml; Survival time, months.

PSA, prostate-specific antigen; SD, standard deviation.

**Table III:** Associated baseline factors of overall survival by univariate and multivariate analysis.

	Univariate			Multivariate		
		
Baseline characteristic	HR	95% CI	P-value	HR	95% CI	P-value
Age, years	0.994	(0.95–1.04)	0.781	1.017	(0.96–1.08)	0.577
Cycles, n	0.741	(0.60–0.92)	0.006	0.704	(0.53–0.93)	0.015
Hemoglobin, g/dl	0.985	(0.75–1.30)	0.912	1.086	(0.75–1.57)	0.662
PSA, ng/ml	1.000	(1.00–1.00)	0.872	1.000	(1.00–1.00)	0.936
PSA nadir, ng/ml	1.002	(1.00–1.01)	0.243	1.002	(1.00–1.01)	0.402
Time to PSA nadir, months	0.736	(0.59–0.91)	0.005	0.677	(0.49–0.94)	0.019

HR, hazard ratio; CI, confidence interval; PSA, prostate-specific antigen. P<0.05 was considered to indicate a statistically significant result.

**Table IV:** PSA kinetics in patients with flare-up phenomenon.

Patient	PSA at baseline, ng/ml	PSA at peak, ng/ml	Time to decline, weeks	Nadir, ng/ml
1	74.38	80.39	5	56.41
2	319.39	342.03	5	97.97

PSA, prostate-specific antigen.
